# Dental Wounds

**Published:** 1900-04-15

**Authors:** 


### DENTAL WOUNDS.

Dentists delight to call themselves dental surgeons, but, unlike the general surgeon, they too often allow open wounds to go uncared for. No surgeon would leave a wound such as is made by the extraction of a tooth expecting it to heal without care and observation. Presumably, most teeth are extracted because their roots are diseased, consequently it should be expected that the socket also would be in a diseased condition. Often the abscess sac is torn away from the end of the root and remains in the socket. Again, carious or often necrotic bone may be present We should not expect the soft tissues of the gum to heal over a place of this character. The dripping of the pus from the socket causes an infection at the orifice with the result that we have sloughing of the gum tissues. As I do not administer gas, I send those of my patients who may require extraction to a specialist, but they are invaribly directed to report to me on the following day. The socket is then thoroughly cleansed, necrotic soft tissues being removed with a curette, and a thorough examination being made for carious or necortic bone. In simple language, the socket is thoroughly cleansed until nothing but healthy tissues remain, whereupon irrigation with an antiseptic followed by an antiseptic dressing suffices at that visit. The case, however, is observed during the week in order to be sure that all diseased parts have been thoroughly removed and healing proceeds.—*Editor Items of Interest.*

